# Longer ice-free seasons increase the risk of nest depredation by polar bears for colonial breeding birds in the Canadian Arctic

**DOI:** 10.1098/rspb.2013.3128

**Published:** 2014-03-22

**Authors:** Samuel A. Iverson, H. Grant Gilchrist, Paul A. Smith, Anthony J. Gaston, Mark R. Forbes

**Affiliations:** 1 Department of Biology, Carleton University, Ottawa, Ontario, Canada; 2 Environment Canada-National Wildlife Research Centre, Ottawa, Ontario, Canada

**Keywords:** arctic, bird, climate change, foraging, polar bear, predator–prey dynamics

## Abstract

Northern polar regions have warmed more than other parts of the globe potentially amplifying the effects of climate change on biological communities. Ice-free seasons are becoming longer in many areas, which has reduced the time available to polar bears (*Ursus maritimus*) to hunt for seals and hampered bears’ ability to meet their energetic demands. In this study, we examined polar bears’ use of an ancillary prey resource, eggs of colonial nesting birds, in relation to diminishing sea ice coverage in a low latitude region of the Canadian Arctic. Long-term monitoring reveals that bear incursions onto common eider (*Somateria mollissima*) and thick-billed murre (*Uria lomvia*) nesting colonies have increased greater than sevenfold since the 1980s and that there is an inverse correlation between ice season length and bear presence. In surveys encompassing more than 1000 km of coastline during years of record low ice coverage (2010–2012), we encountered bears or bear sign on 34% of eider colonies and estimated greater egg loss as a consequence of depredation by bears than by more customary nest predators, such as foxes and gulls. Our findings demonstrate how changes in abiotic conditions caused by climate change have altered predator–prey dynamics and are leading to cascading ecological impacts in Arctic ecosystems.

## 1. Introduction

Climate change can influence species directly by modifying their physical environment or indirectly by altering interactions among organisms [1]. Altered interactions include increased or diminished interspecific competition [2,3], modified host–parasite relationships [4,5], and changes to predator–prey dynamics [6,7]. Rapid environmental changes affecting top predators are of particular relevance because behavioural changes on the part of predators have the potential to restructure food webs and lead to cascading ecological impacts on prey populations [6,8].

To date, the effects of climate change have been most pronounced in northern polar regions where temperatures have risen at a much faster rate than in other regions of the globe [9,10]. The polar bear (*Ursus maritimus*) is an apex predator in the circumpolar Arctic that relies on sea ice as a platform to hunt seals and other marine mammals. The progressively earlier break-up of annual sea ice in low latitude regions of the Arctic has reduced the amount of time available to bears to hunt for seals and amass the fat reserves they require to sustain themselves on shore during the ice-free season [11–14]. Earlier sea ice clearance is a major conservation concern for polar bears because it has been associated with deteriorating body condition, reduced demographic performance and population decline [15–17].

Polar bears are opportunistic predators that feed on a diversity of resources when onshore, including human garbage [18], large terrestrial mammals [19], fish [20] and vegetation [21]. In recent years, ornithologists at a number of Arctic monitoring stations have reported increased numbers of encounters with polar bears coming ashore to feed on bird eggs and more rarely adults or chicks [22–25]. Although it has been suggested that the consumption of terrestrial prey could offset nutritional shortfalls experienced by bears as a consequence of climate change [26], this assertion has been met with scepticism [27]. Resources other than marine mammals are generally regarded as too dispersed and inefficient for bears to consume and digest to be of tangible benefit at a population level. However, considerable uncertainty remains with respect to the frequency and impact of terrestrial foraging by polar bears, including how the prevalence of this behaviour relates to changing environmental conditions and what influence it might have on the fitness and long-term viability of prey populations unaccustomed to intensive depredation by bears.

In this study, we evaluate nest depredation by polar bears on northern common eiders (*Somateria mollissima borealis*) and thick-billed murres (*Uria lomvia*) in a low latitude region of the Canadian Arctic. We use observations from two long-term bird-monitoring stations to assess changes in the frequency of bear incursions and supplement these data with information collected in replicated surveys of eider colonies distributed over a large geographical area. We investigate whether the frequency of bear incursions onto bird colonies is correlated with sea ice conditions and ask whether nest depredation has increased as a result. In addition, we estimate bear prevalence on eider colonies in relation to colony and landscape attributes and compare the magnitude of egg loss due to depredation by bears to that of more customary nest predators such as foxes (*Vulpes* spp.) and gulls (*Larus* spp.). We interpret our findings in relation to their consequences for avian productivity and ecological factors heightening or moderating predation risk.

## 2. Material and methods

### (a) Study system

**Figure 1.**
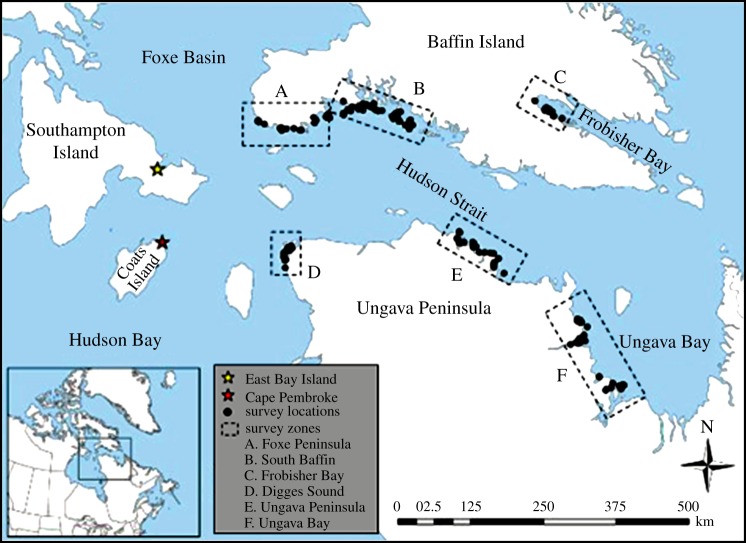
Map of the study area.

We conducted our research in the Hudson Strait–Northern Hudson Bay Narrows region of the Canadian Arctic (figure 1). Sea ice melts completely or nearly so during summer in this region requiring bears to spend several months on land surviving on stored fat reserves and what terrestrial prey they might encounter [28]. It is an area of overlap between the ranges of the Davis Strait and Foxe Basin polar bear subpopulations [29], where ringed seals (*Pusa hispida*) are polar bears’ primary prey item [30].

The region is also within the core breeding range of the northern common eider [31] and thick-billed murre [32]. Northern common eiders breed in colonies that number from a few to several thousand pairs. Clutches average three to five eggs and nesting occurs primarily on small (0.1–5.0 km^2^), near shore (less than 5 km) islands. Thick-billed murres lay a single egg and nest on cliff ledges in colonies comprised thousands to hundreds of thousands of breeding pairs. Both species initiate nests in late June or early July, with incubation periods ranging from 26 to 33 days for eiders and murres, respectively.

### (b) Temporal variation in bear observations in relation to ice conditions

Information about temporal changes in the frequency of polar bear incursions onto bird colonies was obtained from two locations. East Bay Island (64.03° N, 81.79° W), situated off the eastern coast of Southampton Island, Nunavut, supports approximately 5000 common eider breeding pairs and is the largest known eider colony in the Canadian Arctic (see electronic supplementary material, figure S1). Cape Pembroke (62.79° N, 82.28° W), located on the northeastern tip of Coats Island, Nunavut, provides nesting habitat for approximately 30 000 thick-billed murres (see electronic supplementary material, figure S2). Biologists were present at East Bay Island during eider incubation (18 June–1 August) from 1997 to 2012 and at Cape Pembroke during murre incubation (10 July–10 August) from 1988 to 2011 (see electronic supplementary material, table S1). At both locations, bear observations were recorded in daily log books. We used these data to estimate the cumulative number of days on which one or more bears was present each year [TotalBear] and to calculate daily probabilities of bear presence [BearDay] (see the electronic supplementary materials, appendix A: *Polar bear observations at East Bay Island and Cape Pembroke*).

Sea ice coverage information was obtained using the Canadian Ice Service's IceGraph Tool 2.0 (http://dynaweb.cis.ec.gc.ca/IceGraph20) [33]. We queried the database for weekly estimates of the proportion of the sea surface covered by ice (IceCT) in Northern Hudson Bay Narrows, which encompasses both East Bay Island and Cape Pembroke. To facilitate our analyses, we classified sea ice habitat in relation to its quality for polar bears, where less than 30% IceCT is considered non-habitat, 31–60% IceCT is poor habitat and greater than 60% IceCT is good or best quality habitat [34,35]. We then derived a series of ice condition indices [IceCover] to characterize winter ice season length and the timing of spring break-up at good-to-poor and poor-to-non-habitat thresholds, as well as IceCT on specified dates corresponding to eider and murre nesting phenology (18 June, early incubation; 2 July, mid-incubation; 16 July, late-incubation; see the electronic supplementary materials, appendix A: *Sea ice conditions*).

When assessing the influence of a climatic factor (e.g. ice coverage) on a biological process (e.g. bear incursions) the co-occurrence of temporal trends can lead to spurious correlations. This is because of the possibility that the trend in biological process results from a relationship with an overlooked casual factor rather than a relationship with the climate factor itself [36]. We predicted a negative association between ice coverage and bear incursions and required a method to control for year-effects. Therefore, in addition to standard regressions of TotalBear and the various IceCover indices against Year, and against each other, we derived IceCover–Year residuals and used the resulting detrended variable [IceCover′] to assess statistical relationships.

We evaluated the data using generalized linear mixed models (GLMMs) [37], which we implemented using the package lme4 in R [38]. We specified BearDay as a binary response variable (binomial distribution; logit link; scalar function: nAQR = 7) and classified year as a categorical random effect [YearRE] to account for the non-independence of daily observations within years. Fixed effect covariates included Year (classified as a continuous variable), Day of the breeding season, Day^2^ and the best fitting representation of detrended IceCover′ from the regressions described above. Data for East Bay Island and Cape Pembroke were pooled and we included Site as a categorical fixed effect. Our global model included YearRE in additive combination with these fixed effects, as well as a Day × Site interaction. We then compared the global model to a series of reduced models, the most basic of which was a YearRE-only model that served as the null model in our candidate set. Model selection was based on maximum-likelihood methods, evidence ratios and Akaike's information criterion corrected for sample size (AICc) [39]. Regression coefficients (i.e. *β*-values on a logit scale) were estimated by model averaging and statistical significance was judged on the basis of sign (positive or negative relationship between the predictor and response variables) and the precision of estimates (wherein values with 95% confidence intervals that did not overlap with zero were considered important predictors).

### (c) Extent and magnitude of nest depredation by polar bears

To examine the extent and magnitude of nest depredation by polar bears in relation to colony and landscape attributes, we used data collected in boat-based surveys of common eider colonies on the south coast of Baffin Island (Nunavut) and the north coast of Québec (Nunavik). The surveys were carried out over the course of three summers during mid- to late-incubation (10–26 July 2010, 6–19 July 2011 and 8–21 July 2012) and encompassed more than 1000 km of coastline divided among six survey zones (figure 1). Islands in the Foxe Peninsula and South Baffin survey zones were visited in multiple years, whereas islands in the other four survey zones were visited on a single occasion (see electronic supplementary material, table S2).

Each island was investigated by three to eight fieldworkers, who walked 10–25 m apart from each other and made successive linear sweeps of all available nesting habitat [40]. When an eider nest was encountered, its status was recorded as either *active*—a nest attended by an incubating hen, or containing eggs or newly hatched ducklings, or *empty*—a nest in which fresh feather down was present but there was no hen, eggs or ducklings. Nests were detected easily because there is little vegetation in our study area and current year breeding attempts could be distinguished from previous year attempts by the presence of fresh feather down [31,40].

We also recorded evidence for the presence of species known to depredate eider eggs, including gulls (principally herring and glaucous gulls (*L. smithsonianus* and *L. hyperboreus*, respectively)), foxes (Arctic (*V. lagopus*) and red (*V. vulpes*)) and polar bears. Predator sign near a nest can be a poor indicator of the species responsible for a particular depredation event because predator species often differ in their detectability. Therefore, we evaluated predator presence at a colony level in relation to visible sign, as opposed to more circumstantial evidence of egg consumption by predators at specific nests. We regarded seeing a predator species as direct evidence of presence, whereas finding animal sign, including faeces, tracks and fur was regarded as indirect evidence (see the electronic supplementary materials, appendix A: *Extent and magnitude of nest depredation by polar bears*).

For our analysis, we summarized the number and proportion of islands with either direct or indirect evidence of predator presence by species group [BearSign, FoxSign, GullSign]. We then used GLMMs to assess variation in the probability of encountering sign (direct and indirect combined) in relation to colony and landscape attributes. GullSign was encountered on nearly all islands (approx. 95%); therefore, we did not evaluate statistical relationships for gulls. In our models, island was treated as a categorical random effect [IslandRE] to control for sampling at some locations in multiple years. Fixed effect covariates included the number of eider nests [sqrtNests], island size [Area], distance to the mainland [Mainland], maximum inter-step distance over open water that a mammalian predator coming from the mainland would have to cross to reach the island [Crossing], distance to the nearest Inuit community if travelling by boat [Village] and ice concentration within a 25 km radius of the island on 25 June [Ice25] (see the electronic supplementary materials, appendix A: *Landscape attributes*). Our candidate set of models included all additive combinations of the predictor variables, as well as an IslandRE-only null model. To avoid over-parametrization, we did not evaluate interactions between variables.

We also estimated the proportion of nests remaining active at the time of survey [NestSuccess] in relation to the types of predator sign encountered on islands [PredatorType: none, gull only, gull and fox, gull and bear], as well as survey zone [Zone], nest abundance [sqrtNests] and date of survey [Date]. We again used GLMMs and treated island as categorical random effect [IslandRE]. We used our analyses to determine colony and landscape attributes associated with documented predator presence and to estimate rates of nest loss when different combinations of predator sign were encountered.

## 3. Results

### (a) Temporal variation in ice conditions and bear observations

The number of days on which biologists observed one or more polar bears during the bird nesting season increased markedly at both the East Bay Island common eider colony (see electronic supplementary material, figure S3*a*) and the Cape Pembroke thick-billed murre colony (see electronic supplementary material, figure S3*b*). Five-year running averages (±s.e.) indicated a sevenfold increase in bear incursions at East Bay Island (TotalBear_1997−2001_ = 1.8 ± 0.5 days with bear × yr^−1^; TotalBear_2008–2012_ = 12.6 ± 0.9 days with bear × yr^−1^) and an eightfold increase at Cape Pembroke (TotalBear_1988–1992_ = 1.4 ± 0.5 days with bear × yr^−1^; TotalBear_2007–2011_ = 12.0 ± 1.4 days with bear × yr^−1^), respectively.

**Figure 2.**
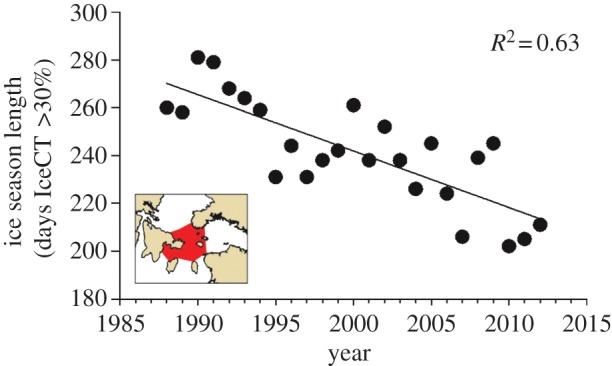
Decline in annual sea ice coverage in Northern Hudson Bay Narrows, Canada from 1988 to 2012 quantified in relation to the number of days that more than 30% of the sea surface was covered by ice each winter. (Online version in colour.)

Sea ice coverage declined dramatically in Northern Hudson Bay Narrows during our study interval. We estimated a 2.4 (±0.4) d × yr^−1^ decrease in ice season length at the 30% IceCT threshold (poor to non-habitat) from 1988 to 2012 (figure 2), which equates to a two month loss of sea ice habitat for polar bears. Annual estimates for ice season length ranged from a maximum of 281 days in 1990 to minimum of 202 days in 2010 (see electronic supplementary material, table S3). A similar trend was evident for ice season length at the 60% IceCT (good to poor habitat). Advancing spring break-up date accounted for approximately half of the sea ice loss at both the 30 and 60% thresholds. Ice coverage at specified dates (18 June, 2 July and 16 July) corresponding with eider and murre nesting phenology (early, mid- and late-incubation) also exhibited sharp declines; however, the fixed date indices exhibited greater inter-annual variability than the indices calibrated in relation to duration of ice coverage or break-up date (see electronic supplementary material, table S3). Among the IceCover indices that we evaluated, season length at the 30% IceCT threshold (*SeasonLength30*) was the most strongly correlated with bear incursions on both East Bay Island and Cape Pembroke (see electronic supplementary material, tables S4 and S5). Therefore, we used *SeasonLength30* as our index of IceCover in subsequent analyses.

The best fitting model in our candidate set of GLMMs investigating variation in daily probability of bear incursions [BearDay] indicated that Year, Day, Day^2^ and detrended IceCover′ were significant predictors variables (log(*L*) = −507.67, *K* = 6, AIC_c_ = 1027.40. *w_i_* = 0.72). The global model, which included the same predictors, as well as Site and Site × Day was also supported by the data (log(*L*) = −506.70, *K* = 8, ΔAIC_c_ = 2.12. *w_i_* = 0.25) (see electronic supplementary material, table S6).

**Figure 3.**
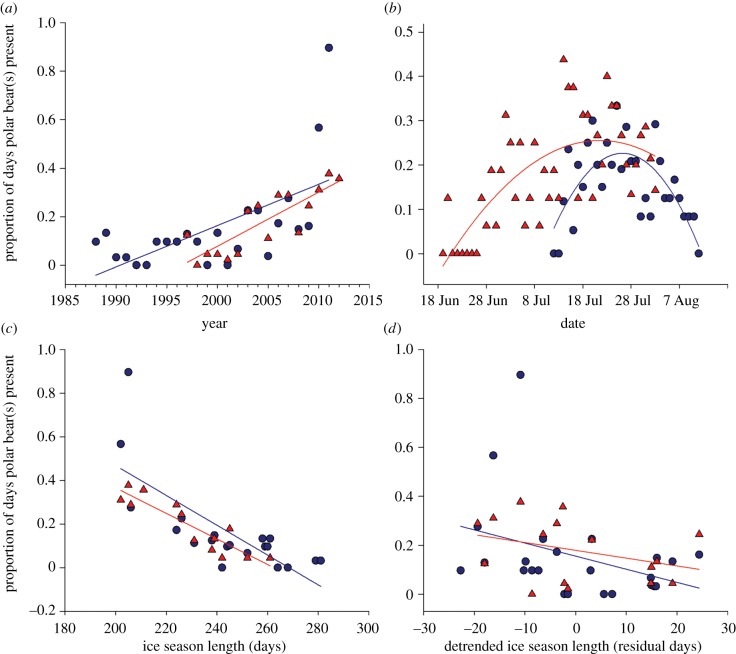
Summary proportions for the frequency with which one or more polar bears was observed during the nest incubation period [BearDay] at the East Bay Island common eider colony (red symbols) and Cape Pembroke thick-billed murre colony (blue symbols) in relation to (*a*) year (*b*) day of the breeding season, (*c*) ice season length (number of days IceCT more than 30%) and (*d*) detrended ice season length as determined by residual regression.

Model averaged regression coefficients indicated an increasing probability of bear incursions with advancing year (*β*_Year_ = 0.152, 95% CI: 0.111–0.192) (see electronic supplementary material, table S7), which was reflected in raw data plots illustrating summary proportions parsed by site and fit with a linear model (figure 3*a*; electronic supplementary material, table S8). We also estimated an increasing probability of bear incursions with advancing date during the incubation period (*β*_Day_ = 0.221, 95% CI: 0.145–0.297), but a declining probability late in the breeding season when eggs begin to hatch or are lost to predators (*β*_Day_^2^ = −0.003, 95% CI: −0.004 to −0.002). Summary proportions fit with a quadratic equation indicated that bear incursions peaked in mid-July during late-incubation (figure 3*b*; electronic supplementary material, table S8). We also found that the daily probability of bear incursion was higher on East Bay Island than Cape Pembroke (*β*_Site_ = 0.382, 95% CI: 0.097–0.669); however, we estimated a negative interaction between site and day, suggesting later arrival and a greater rate of increase with advancing date at Cape Pembroke (*β*_Site×Day_ = −0.011, 95% CI: −0.023 to −0.001). In accordance with the main prediction of our study, we estimated a negative association between bear incursions and detrended ice cover (*β*_IceCover′_ = −0.034, 95% CI: −0.051 to −0.016; figure 3*c,d*).

### (b) Extent and ecological correlates

Our broad-scale geographical sampling conducted in 2010 through 2012 included 230 islands supporting more than 32 500 common eider nesting pairs (see electronic supplementary material, table S2). These years coincided with the three lowest early summer ice coverage extents on record in Northern Hudson Bay Narrows and three of the four lowest ice coverage extents on record for Hudson Strait. However, variation in ice coverage was apparent among locations and across years, with 2012 being a relatively heavy ice year (IceCT > 70% on 25 June) in the Foxe Peninsula, South Baffin and Frobisher Bay survey zones (see electronic supplementary material, figure S4).

During the course of our surveys, we observed 22 polar bears (including four cubs) on 16 islands with eider colonies. Indirect evidence for the presence of bears was observed on a further 63 islands and polar bear presence (direct + indirect evidence) was estimated on 34% of islands in total (table 1). We observed two foxes and encountered indirect evidence of fox presence on a further 24 islands (combined prevalence 11%). Gulls were observed on or flying above 160 islands and indirect evidence of gull presence was noted on a further 58 islands (combined prevalence 95%).

**Table 1.** Per cent of islands containing common eider nesting colonies on which either direct or indirect evidence of polar bear, fox or gull presence was detected during surveys in the Hudson Strait–Northern Hudson Bay Narrows region of the Canadian Arctic during July 2010, 2011 and 2012.

species group	type of evidence	description of evidence	no. islands	per cent of islands^a^ %
polar bear	total	all	79	34.3
	direct	live bear (*n* = 22)	16	7.0
	indirect	faeces	31	13.5
		tracks or digging	6	2.6
		fur	2	0.9
		depredated nests with direct sighting or faeces within a 1 km radius	24	10.4
fox	total	all	26	11.3
	direct	live fox (*n* = 2)	2	0.9
	indirect	active den	1	0.4
		faeces	5	2.2
		fur	3	1.3
		depredated nests with direct sighting or faeces within a 1 km radius	15	6.5
gull	total	all	218	94.8
	direct	live gull	160	69.6
	indirect	depredated nests with direct sighting within a 1 km radius	58	25.2

^a^Number of islands with indicated evidence of predator presence divided by the total number of islands surveyed (*n* = 230 islands).

Our GLMM examining variation in the prevalence of BearSign in relation to colony and landscape attributes indicated that nest abundance, distance to the mainland, distance to the nearest village and ice conditions were important predictor variables (log(*L*) = −117.74, *K* = 6, AIC*c* = 247.86, *w*_i_ = 0.33); however, several other models also were supported by the data (ΔAIC*c* < 4; electronic supplementary material, table S9). In accordance with *a priori* predictions, model averaged regression coefficients indicated a negative association between BearSign and ice concentration (*β*_Ice25_ = −0.534, 95% CI: −0.077 to −0.030) and positive associations with nest abundance (*β*_sqrtNests_ = 0.091, 95% CI: 0.047–0.136), distance to mainland (*β*_Mainland_ = 0.051, 95% CI: 0.005–0.097) and distance to the nearest village (*β*_Village_ = 0.012, 95% CI: 0.004–0.020; electronic supplementary material, table S10).

**Figure 4.**
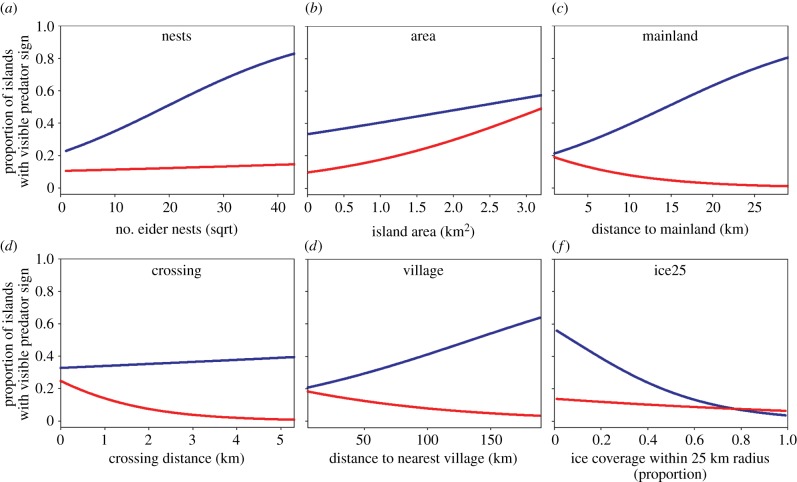
Probability of encountering polar bear sign (blue lines) or fox sign (red lines) on islands with common eider colonies in relation to (*a*) the number of eider nests, (*b*) island size, (*c*) distance to the mainland, (*d*) crossing distance at low tide, (*e*) distance to the nearest Inuit village and (*f*) sea ice concentration at the onset of incubation (25 June) within a 25 km radius of the island.

With respect to FoxSign, several models had a similar fit to the data (see electronic supplementary material, table S11). Multi-model inference and model averaging indicated that the probability of encountering fox sign increased with island size (*β*_Area_ = 0.224, 95% CI: 0.082–0.366) and decreased with crossing distance (*β*_Crossing_ = −1.486, 95% CI: −2.922 to −0.050). Regression coefficients could not be distinguished from zero for the other variables included in our analysis (see electronic supplementary material, table S12). Logistic regression of pair-wise relationships between the predictors and response variable illustrates patterns for both bears and foxes within the range of conditions that we sampled (figure 4).

Bears were observed to consume eggs on multiple occasions and the combined effects of bear predation and gull predation caused near total reproductive failure in several instances. For example, we surveyed an island east of Cape Dorset (64.06° N, 73.53° W) on 11 July 2011. We counted 536 eider nests, among which 334 were active. Fresh bear faeces containing egg shell fragments were found on the island and we counted greater than 30 gulls, many of which were actively hunting eider eggs. We returned to the same island 2 days later and observed a bear on the island eating eggs and more than 50 gulls present. We resurveyed the colony after the bear left of its own accord and counted 24 active nests. This example was not unique, but was among the most definitive cases of near total nest destruction on a colony.

The proportion of nests remaining active at the time of survey, which was our index of NestSuccess, varied substantially in relation to the types of predator sign encountered, as well as nest abundance and survey date (log(*L*) = −38.49, *K* = 7, AIC*c* = 91.50, *w*_i_ = 0.68) (see electronic supplementary material, table S13). Model averaged regression coefficients indicated lower nest success on islands with both gull and bear sign (*β*_Gull and Bear_ = −2.612, 95% CI: −3.962 to −1.254) or both gull and fox sign (*β*_Gull and Fox_ = −1.772, 95% CI: −3.251 to −0.285) compared to islands with gull sign alone (*β*_Gull_ = −0.058, 95% CI: −1.787 to 0.637; electronic supplementary material, table S14). The proportion of nests remaining active decreased with advancing date (*β*_Date_ = −0.091 95% CI: −0.148 to −0.035) as our surveys were conducted during incubation and simultaneous with the arrival of nest predators. We also estimated positive association between nest success and nest abundance within a colony (*β*_sqrtNests_ = 0.043, 95% CI: 0.004–0.081) despite the fact the bear sign was positively correlated with nest abundance. Models including a survey zone effect were not well supported by the data. Raw data summaries were consistent with modelled results. We estimated that nest success (±s.e.) was three times lower on islands with both bear and gull sign (proportion of nests surviving to the date of survey = 0.22 ± 0.01) compared to islands with gull sign alone (0.66 ± 0.01) and was roughly 1.5 times lower on islands with both fox and gull sign (0.39 ± 0.02) compared with islands with gull sign alone (table 2).

**Table 2.** Proportion of nests remaining active (nest success) in relation to the types of predator sign found on islands containing common eider colonies.

type of predator sign	islands surveyed	total nests	nest success (±s.e.)
no predator sign	15	183	0.72 (±0.03)
gull only	114	14 416	0.66 (±0.01)
fox and gull	22	3041	0.39 (±0.02)
bear and gull	75	14 441	0.22 (±0.01)
bear, fox and gull	4	511	0.06 (±0.01)
total	230	32 592	0.44 (±0.01)

## 4. Discussion

Predictions about biological responses to climate change focus largely on the environmental tolerances of individual species [41]. The circumpolar Arctic is experiencing major, ongoing reductions in both annual and multi-year sea ice [42], with low latitude regions being most impacted [43]. Hudson Strait and Northern Hudson Bay Narrows, where our study was conducted, are within a region of the Canadian Arctic that has undergone a nearly two month reduction in annual ice cover over the past three decades [34]. Substantial open water areas are now routinely encountered in May and the near shore seasonal ice environment upon which polar bears depend has been drastically altered.

Changing climatic conditions can also lead to shifts in interspecific interactions that influence population and community dynamics [6]. Depredation of colonial nesting bird eggs by polar bears is not a new phenomenon; indeed, documented accounts date back more than a century [44]. However, there is considerable evidence to suggest that bears are becoming more frequent visitors to bird colonies during the nesting season [22–25] and we demonstrated that the frequency of bear visits is negatively correlated with sea ice coverage. Ice-free seasons are not only becoming longer, but freeze-up and break-up dates are also becoming more variable and we measured remarkable similarity in bear attendance patterns at widely separated locations involving diverse avian species. We interpret our findings as evidence for an environmentally driven shift, wherein prey of comparatively low energetic value (bird eggs) are actively sought by polar bears when ecological conditions (lack of sea ice) prevent them from acquiring sufficient energy reserves from their preferred prey item (seals).

The negative association between bear incursions and sea ice conditions that we documented held after controlling for co-occurring temporal trends, thus constituting strong support for a causal effect of ice coverage on bears’ decision to consume eggs. However, year-effects remained evident, suggesting that variables not included in our models also influenced observed patterns. For example, increasing polar bear numbers or declining seal numbers could contribute to the increased prevalence of bears on bird colonies. Available data are insufficient to estimate polar bear [29] population trends with adequate precision in our study area; however, there is no evidence to support an increase in bear population size approaching the magnitude of increase that we documented in bear visits at East Bay Island and Cape Pembroke. More plausible factors to consider include increased duration of stay by individual bears on bird colonies and a tendency for bears once having discovered a resource to return in subsequent years and pass information from mother to offspring.

Broad-scale geographical sampling of common eider colonies during years of record low ice coverage indicate that nest depredation by polar bears is extensive and that when bears raid eider colonies nesting success is severely reduced. Population modelling suggests that the species’ population growth rate is more sensitive to variations in adult survival than variations in annual productivity; however, retrospective analyses based on field data indicate that survival rates have proved relatively invariant over time, whereas fluctuations in reproductive success have been a primary driver of changes in population size [45,46]. Climate-influenced variation in rates of nest depredation strongly influence eider population dynamics [47] and given the magnitude of egg loss that we observed on heavily depredated colonies, local extirpation would be expected within only a few generations if it were to continue unabated. However, predicting the population-level response of eiders, as well as other Arctic-nesting birds, to increased nest depredation by polar bears requires multiple considerations.

We measured considerable spatial- and habitat-related variation in bear prevalence on eider colonies. Eiders nesting on islands nearer to shore and in closer proximity to Inuit villages were less vulnerable to depredation by bears than eiders nesting on islands further from the mainland and nearer seal hunting grounds. In addition, eiders nesting solitarily or in small aggregations were largely ignored by bears. These observations suggest that distributional and density-dependent factors set a lower threshold for bears’ interest in eider eggs that we predict will moderate population impact.

While ground nesting species such as common eiders may be particularly vulnerable to nest depredation by polar bears; bears have also proved an emerging conservation concern for cliff nesting thick-billed murres. Gaston & Elliot [48] attributed breeding failure of up to 30% of the murre population at Cape Pembroke to polar bears during years of high bear visitation. In the case of murres, egg loss resulted from a combination of direct depredation and as a by-product of murres’ fleeing from bears and in the process dislodging their own eggs. However, the steepest and narrowest cliff ledges located in the core of the colony have remained largely unaffected by bears. Thus, murres appear less susceptible than eiders to large-scale population reduction owing to differences in nesting habitat.

Relationships between changing ice conditions and access to eggs by different predator species are also potentially important. Foxes are traditional nest predators for eiders and eiders nest on islands in large part to minimize fox depredation [31]. In this study, we estimated a greater prevalence of bear sign (34%) than fox sign (11%) on eider colonies; however, our results pertain only to visible sign and the species probably differ in their detectability. Foxes tend to cache eggs and we observed fewer fox tracks and faeces on the islands where such signs were encountered than was the case for polar bears, which were observed to destroy large numbers of eggs and left abundant sign. Unresolved issues with detectability notwithstanding, we believe our results reflect biological reality. Advancing spring ice melt has been associated with reduced access to islands by foxes [47]. Open water is a barrier for foxes, whereas swimming a distance more than 1 km to reach an eider colony is not a significant impediment for a polar bear. Thus, the same forces exacerbating predation risk from bears appears to be reducing predation risk from foxes.

Our results demonstrate how the direct effects of sea ice loss on polar bears are having unanticipated indirect effects on breeding birds. While the nutritional benefits to bears are not known, our results clearly demonstrate that nest depredation is not limited to a few bears or a handful of nests. Our results are consistent with assertions that polar bears are experiencing difficulty meeting their energetic demands in locations where ice-free seasons have grown significantly longer. Perhaps most importantly, they highlight the importance of incorporating interspecific interactions into predictions about the ecological impacts of changing environmental conditions in a rapidly warming Arctic.
